# A unified machine-learning framework for ab initio multiscale modeling of liquids

**DOI:** 10.1073/pnas.2610049123

**Published:** 2026-07-24

**Authors:** Anna T. Bui, Stephen J. Cox

**Affiliations:** ^a^ Yusuf Hamied Department of Chemistry, University of Cambridge, Cambridge CB2 1EW, United Kingdom; ^b^ Department of Chemistry, Durham University, Durham DH1 3LE, United Kingdom

**Keywords:** machine-learned interatomic potentials, classical density functional theory, first-principles modeling, phase transition, supercritical fluids

## Abstract

Understanding and predicting the behavior of liquid matter across length scales—using only the microscopic interactions encoded in the Schrödinger equation—remains a central challenge in the physical sciences. Achieving this goal requires not only an accurate and efficient description of intermolecular forces but also a consistent framework that bridges the micro-, meso-, and macroscales. Here, by combining machine-learned interatomic potentials (MLIPs) with neural classical density functional theory (cDFT), we present such a framework. MLIPs trained on quantum-mechanical energies and forces are used to generate inhomogeneous density profiles, which then serve as the training data for neural cDFT. The resulting ab initio neural cDFT is more computationally efficient than molecular simulations and provides a conceptually transparent route to the thermodynamics of both homogeneous and planar inhomogeneous systems. We demonstrate the approach for both water and carbon dioxide using several exchange–correlation functionals. Beyond accurately reproducing—at the level of the underlying approximate electronic structure—bulk equations of state and liquid–vapor phase diagrams, ab initio neural cDFT predicts, from first principles, how confinement modifies liquid–vapor coexistence in water. It also captures complex behavior in supercritical carbon dioxide such as the Fisher–Widom and Widom lines. While current applications are limited to bulk fluids and planar geometries, this approach establishes a general first-principles route to multiscale modeling of fluids by unifying two independently developed machine-learning paradigms. This work represents an important step toward generalizing cDFT beyond simple empirical potentials to chemically complex systems.

## Significance

Liquids play a central role across science and technology. Yet predicting their behavior from first principles remains a significant challenge. Quantum mechanical approaches accurately capture molecular interactions but are typically limited to atomistic scales, making it hard to describe emergent phenomena such as liquid–vapor phase equilibria under confinement. We present a machine-learning framework based on first principles that efficiently describes the emergent properties of fluids while retaining molecular resolution. With this framework, we straightforwardly quantify how nanoconfinement influences water’s liquid–vapor coexistence and map structural transitions in supercritical carbon dioxide. By bridging quantum mechanical Hamiltonians and mesoscale behavior, our approach provides a general framework for multiscale modeling of fluids from first principles.

Liquids play a pivotal role across biology, energy storage, catalysis, and environmental science. Their influence ranges from processes at the molecular scale (1–3), where the quantum nature of interatomic interactions is important, to the nano-, meso-, and macroscopic redistribution of the fluid (4–6), such as near phase transitions. Put simply, comprehensively understanding the behavior of liquids is an inherently multiscale problem. A central goal in chemical physics is to predict such multiscale phenomena from first principles, relying solely on knowledge of the underlying microscopic interactions encoded in the Schrödinger equation (7). Any genuine multiscale modeling approach needs to faithfully describe both a system’s intermolecular interactions, and its collective behavior.

Rooted in the majority of existing multiscale frameworks is the notion of different “levels of theory.” For example, in the case of QM/MM (8) a region where interactions are accurately described by quantum mechanics (QM) is embedded within an environment (e.g., solvent) described by a computationally efficient molecular mechanics (MM) model. Alternatively, the results of accurate ab initio calculations can be used to parameterize a coarser description of the system, e.g., by passing reaction energies into a kinetic model (9). While such strategies have proven powerful in contexts such as biochemistry (10) and heterogeneous catalysis (11), how to best combine different levels of theory is not always apparent. In this article, we demonstrate a simple multiscale modeling strategy for liquids that simultaneously describes micro-, meso-, and macroscale phenomena on an equal footing, entirely from first principles.

The multiscale framework that we present unifies two areas of physical science in which machine learning (ML) is playing a transformative role. The first of these is machine-learned interatomic potentials (MLIPs). By providing relatively inexpensive surrogate models of a system’s potential energy surface that would otherwise be obtained from costly electronic structure calculations, MLIPs are vastly increasing the efficiency of molecular simulations of condensed phases. The second is classical density functional theory (cDFT), an exact statistical mechanical framework for inhomogeneous fluids that, in principle, provides mesoscale insight while retaining microscopic resolution (12, 13).

The development of MLIPs is an avenue of research actively pursued by many research groups (14–19), and is sufficiently advanced that predicting microscopic structure, dynamics, and bulk thermodynamics of fluids has essentially become routine (20–25). Yet, while direct simulation using MLIPs to compute emergent properties such as phase diagrams (26–29), interfacial free energies (2), and adsorption equilibria (30) is possible, doing so comes with a significant computational burden. Moreover, such simulations are often sensitive to simulation size, slow dynamics associated with rare events, and largely limited to describing closed systems; standard molecular dynamics (MD) simulations are typically performed with a fixed number of molecules. For systems in equilibrium with a reservoir, as is often the case for adsorption and confinement phenomena, a framework that naturally lends itself to open systems can greatly simplify analysis.

To overcome the intrinsic limitations of molecular simulation, we will also leverage recent advances in cDFT, in which, at temperature T, both a fluid’s equilibrium one-body density ρ(r) and thermodynamics are obtained by minimizing the system’s grand potential functional

[1] $$\begin{array}{ll} \Omega_{V}\left(\left[\rho \right],T\right)= & F_{\text{intr}}^{\text{(id)}}\left(\left[\rho \right],T\right)+F_{\text{intr}}^{\text{(ex)}}\left(\left[\rho \right],T\right)\\ & +\int \text{d}r\rho \left(r\right)\left(V_{\text{ext}}(r)-\mu \right), \end{array}$$

in a single, self-consistent calculation (12, 13). Inhomogeneity in ρ may arise from the external potential $V_{\text{ext}}$, or from the formation of interfaces between phases at coexistence. The intrinsic Helmholtz free energy functional, which is independent of $V_{\text{ext}}$ and the chemical potential μ, comprises an ideal part $F_{\text{intr}}^{\text{(id)}}$ that is known exactly, and an excess part $F_{\text{intr}}^{\text{(ex)}}$ that arises from intermolecular interactions.

In direct analogy to the exchange–correlation (xc) functional in electronic structure (31), the use of cDFT as a practical tool relies upon accurate approximations to $F_{\text{intr}}^{\text{(ex)}}$. Note that, in contrast to the exact xc functional in electronic structure, $F_{\text{intr}}^{\text{(ex)}}$ is not universal; it depends upon the underlying intermolecular potential and T. For a given intermolecular potential, however, $F_{\text{intr}}^{\text{(ex)}}$ is unique. While accurate forms of $F_{\text{intr}}^{\text{(ex)}}$ have been available for hard sphere fluids for decades (32–34), going beyond simple liquids, where hard spheres can act as a suitable reference, has remained a formidable challenge (35–38).

Recent works have shown that ML can be used to learn highly accurate representations of $F_{\text{intr}}^{\text{(ex)}}$ from molecular simulation data (39–42). Specifically, Sammüller et al., in a development dubbed “neural cDFT,” have shown how a neural network can be trained to represent the one-body direct correlation functional (43),

[2] $$\begin{array}{c} c^{\left(1\right)}\left(r;\;\left[\rho \right],T\right)=-\frac{\delta \beta F_{\text{intr}}^{\text{(ex)}}\left(\left[\rho \right],T\right)}{\delta \rho (r)}, \end{array}$$

where $\beta =1/k_{\text{B}}T$ with $k_{\text{B}}$ as the Boltzmann constant. Neural cDFT has been successfully applied to a range of model fluids, including hard spheres, Lennard–Jones (both single- and two-component), primitive electrolytes, and polar fluids (44–48), where it has been shown to describe complex phenomena such as liquid–vapor coexistence, liquid–liquid phase separation, azeotropy, and electromechanical phenomena. All of these previous studies, however, have relied exclusively on empirical interatomic potentials, leaving cDFT’s potential to predict mesoscopic behavior directly from a first-principles Hamiltonian untapped.

**Fig. 1.**
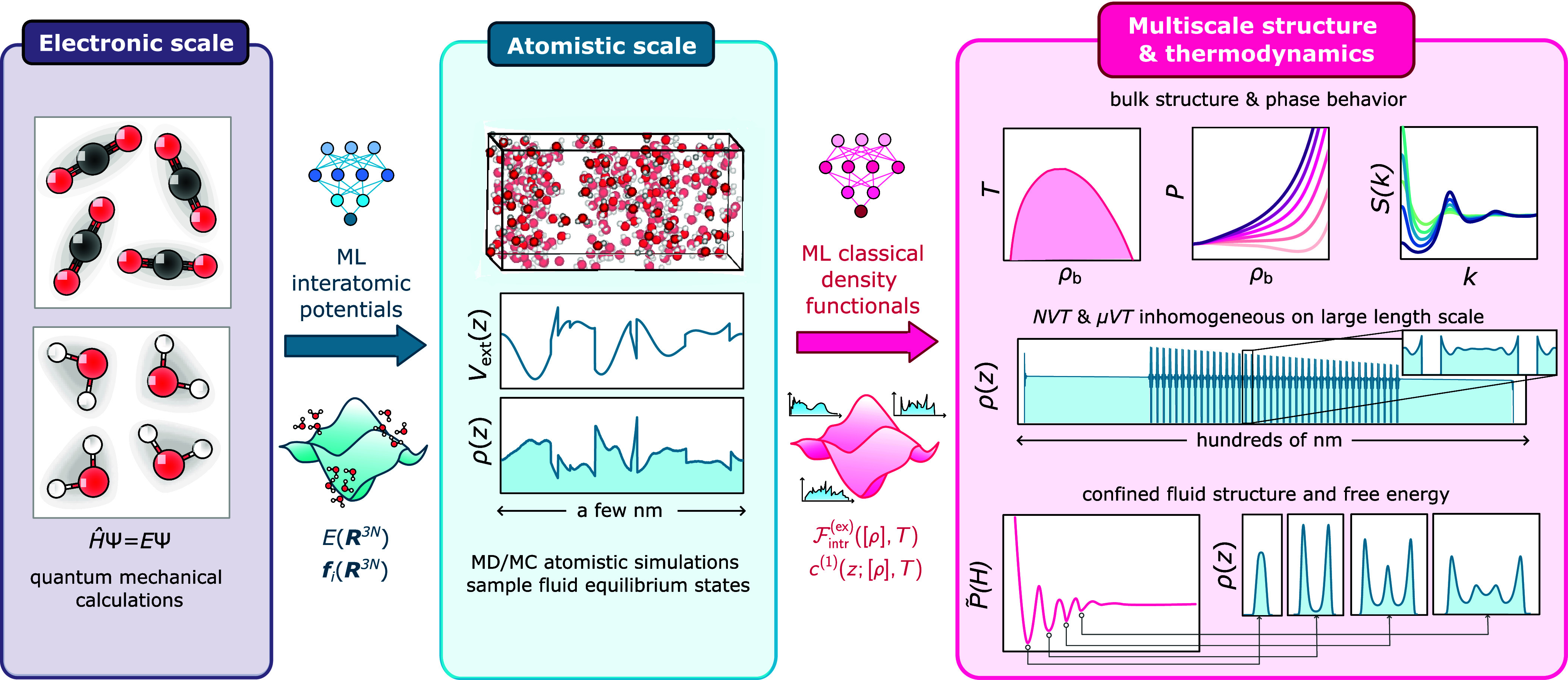
Overview of ab initio neural cDFT. Energies and forces from small-scale electronic structure calculations are used to train an MLIP that represents the potential energy surface, enabling efficient sampling of atomic configurations on nanometer length scales. Equilibrium density profiles obtained from molecular simulations with the MLIP under inhomogeneous external potentials then form the training set for neural cDFT. The resulting ab initio neural cDFT can be used to obtain bulk thermophysical properties, liquid–vapor phase equilibria, and to investigate inhomogeneous systems both on large length scales, and under nanoconfinement.

Here, we fill this gap by presenting an integration of MLIPs trained on quantum-mechanical data into neural cDFT, yielding a genuinely ab initio cDFT for molecular fluids. We demonstrate this framework for two liquids of broad importance: water and carbon dioxide. After validating the resulting ab initio neural cDFT against molecular simulations, we use it to straightforwardly investigate phenomena that would be extremely challenging to obtain with traditional computational approaches. Specifically, we investigate the influence of confinement on water’s liquid–vapor phase behavior, with a clear thermodynamics prescribed by the grand canonical ensemble. We also compute the fluid phase diagram of carbon dioxide, including the structural crossovers encoded by the Fisher–Widom and Widom lines. A schematic overview of our approach is given in Fig. 1.

## Constructing ab initio neural cDFT

Rather than use MLIPs to study the behavior of fluids directly, we instead use their predicted forces to generate equilibrium planar inhomogeneous density profiles $\rho_{\text{eq}}\left(z\right)$ from relatively small simulations. Specifically, for a given T, total number of molecules, and $V_{\text{ext}}\left(z\right)$ (each chosen randomly), we obtain $\rho_{\text{eq}}\left(z\right)$ from an MD simulation. The corresponding one-body direct correlation function is given by the Euler–Lagrange equation that results from the variational principle of cDFT (12)

[3] $$\begin{array}{c} c^{\left(1\right)}\left(z;\;\left[\rho_{\text{eq}}\right],T\right)=\;\ln(\zeta^{-1}\Lambda^{3}\rho_{\text{eq}}\left(z\right))+\beta (V_{\text{ext}}\left(z\right)-\mu ), \end{array}$$

where Λ is the thermal de Broglie wavelength, and ζ is an intramolecular partition function; this is necessary as μ couples to the number of molecules rather than atoms (49). For the remainder of the article, since we only consider the system at equilibrium, we hereinafter drop the “eq” subscript, such that ρ refers to the equilibrium density profile.

In the original implementations of neural cDFT, many grand canonical Monte Carlo (GCMC) simulations, i.e., those with different, but known, $V_{\text{ext}}$ and μ, were used to generate a training set by obtaining $c^{\left(1\right)}\left(z\right)$ directly from Eq. **3**. For each discrete value of $c^{\left(1\right)}\left(z\right)$, treated as the target in the learning procedure, a finite window of neighboring points in the corresponding ρ(z) is passed through a deep neural network to learn the functional dependence on density (43). The temperature dependence can also be established by passing T to the neural network (44). For the MLIPs we use here, however, GCMC is impractical; this is due to both a lack of efficient software packages and the fact that insertion moves in GCMC can lead to configurations far outside the MLIP training set (50). We therefore adopt a recent development whereby training data is obtained from canonical MD simulations (51), in which both $c^{\left(1\right)}$ and the set of chemical potentials of the training simulations are learned by the neural network; this is achieved by treating Eq. **3** itself as the loss function (*Materials and Methods*). Thus, μ is treated as a latent variable in the machine-learning problem.

The neural cDFT procedure that we describe above is limited to learning the one-body direct correlation functional of fluids with planar inhomogeneous geometries, though we stress that the systems remain three dimensional. This limitation is, in part, due to the underlying machine-learning architecture, though we note recent efforts to extend neural cDFT to resolving the fluid density in two dimensions (52). Moreover, even if we were to attempt to adopt the approach described in ref. 52 to full three-dimensional resolution, obtaining reliable training data would currently prove too onerous for the MLIPs we use in this study. It is important to stress, however, that functions of the bulk fluid with radial symmetry, such as the radial two-body direct correlation function, can be obtained by automatic differentiation of $c^{\left(1\right)}\left(z\right)$ from neural cDFT, followed by radial projection (43).

As $F_{\text{intr}}^{(\text{ex)}}$ is not a universal functional, for each fluid and xc functional we must learn $c^{\left(1\right)}$ separately. For water, we therefore learn $c^{\left(1\right)}$ corresponding to the SCAN (53) and RPBE-D3 (54, 55) functionals, as MLIPs for these have previously been successfully applied to liquid–vapor coexistence. For the purposes of comparison, we also present results for TIP4P/2005 (56), a popular empirical potential for studying liquid water. In the case of carbon dioxide, we obtain $c^{\left(1\right)}$ for the PBE-D3 (57), BLYP-D3 (58), and SCAN-rVV10 (59) functionals, along with the well-established TraPPE empirical potential (60). Although rooted in quantum mechanics, all xc functionals we consider are necessarily approximate representations of the true microscopic Hamiltonian. Our selection is guided primarily by the availability of extensive reference data—such as bulk thermodynamic properties and liquid–vapor coexistence—from prior MLIP-based simulation studies, which enables meaningful validation of our approach. While alternative electronic-structure methods could in principle provide more accurate benchmarks (61), the ab initio neural cDFT framework itself is agnostic to this choice, provided that suitable MLIPs can be constructed.

**Fig. 2.**
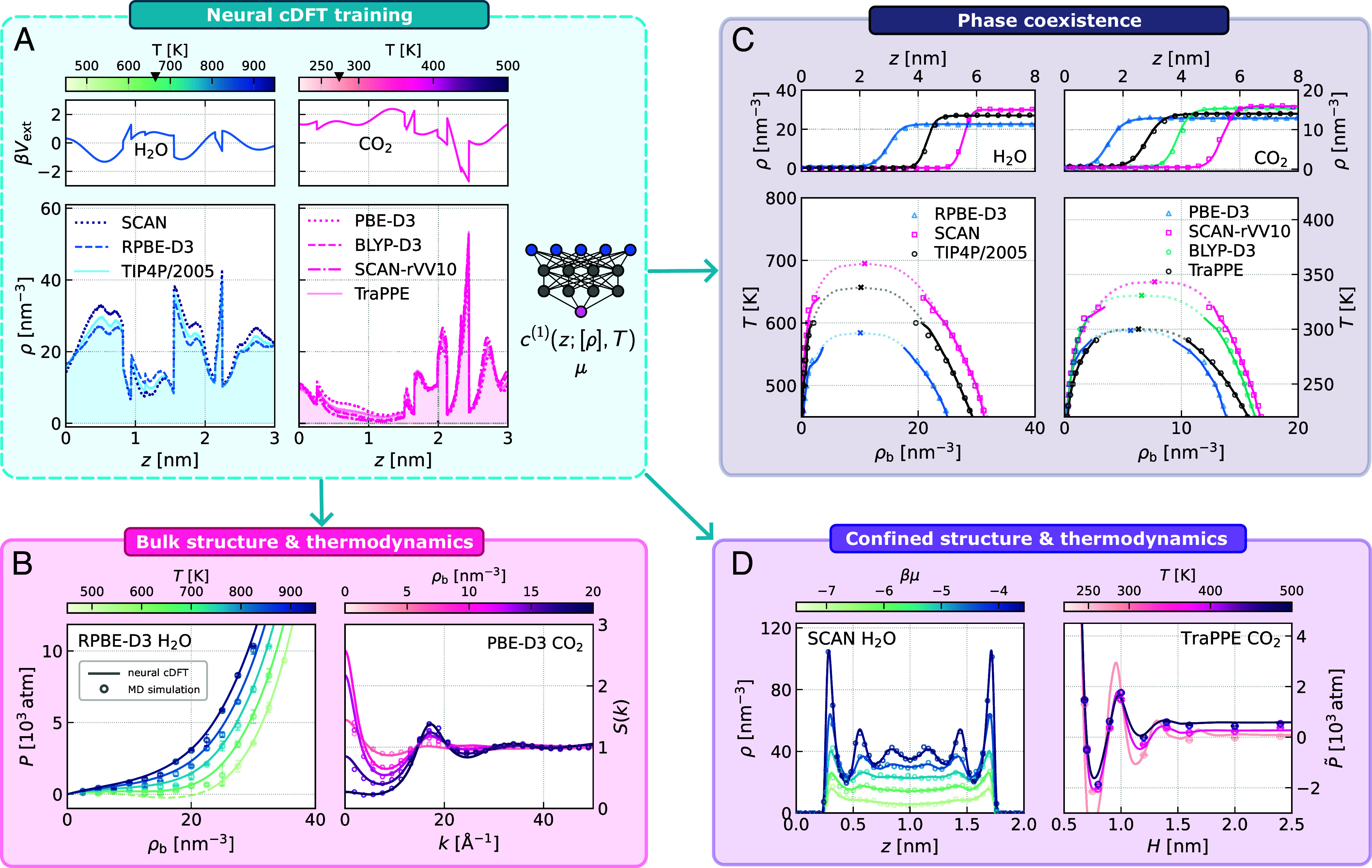
Accurate and efficient description of liquids with ab initio neural cDFT. (*A*) Typical random $V_{\mathrm{ext}}\left(z\right)$ and corresponding ρ(z) used to train the neural cDFT, as obtained from molecular simulations. Results are shown for both water and carbon dioxide, with several interatomic potentials, as indicated in the legends. (*B*, *Left*): P vs. $\rho_{b}$ for RPBE-D3 water along several isotherms. The van der Waals loop for the lowest temperature is indicated by the dashed line. (*B*, *Right*): S(k) for PBE-D3 carbon dioxide for different $\rho_{b}$ at T=360 K. (*C*, *Top*): ρ(z) at liquid–vapor coexistence for water at T=500K (*Left*) and carbon dioxide at T=250K (*Right*), for different interatomic potentials. (*C*, *Bottom*): Liquid–vapor binodals for water (*Left*) and carbon dioxide (*Right*) with the different interatomic potentials, as indicated in the legend. (*D*, *Left*): ρ(z) of SCAN water at different μ confined between graphene sheets. (*D*, *Right*): Effective pressure of TraPPE carbon dioxide at different T, confined between graphene sheets. In (*B*–*D*), symbols show results from MD simulations and solid lines show results from ab initio neural cDFT.

For each xc functional or empirical potential, between 500 and 1,000 training simulations comprising 40–1,024 molecules were performed with different random $V_{\text{ext}}$, and across a broad range of T (*Materials and Methods*). Representative $V_{\text{ext}}$ and ρ(z) used during training are shown in Fig. 2*A*. Taking advantage of the canonical learning procedure, we also include ρ(z) from simulations of liquid–vapor coexistence (comprising either 832 water molecules or 1,536 carbon dioxide molecules), in which $V_{\text{ext}}=0$. We employed either DeepMD (15) or HD-NNP (14) for the underlying MLIP architecture (*Materials and Methods*), however, we show in *SI Appendix*, Fig. S1 that MACE (16) also provides stable dynamics with the random forms for $V_{\text{ext}}$ that we use.

## Validation of Ab Initio Prediction of Structure and Thermodynamics

With a trained neural network representation of $c^{\left(1\right)}$, the equilibrium structure for any given T, $V_{\text{ext}}$, and μ is readily obtained by self-consistently solving the Euler–Lagrange equation (Eq. **3**), rearranged here with ρ as the object:

[4] $$\begin{array}{c} \zeta^{-1}\Lambda^{3}\rho \left(z\right)=\;\exp[-\beta (V_{\text{ext}}\left(z\right)-\mu )+c^{\left(1\right)}\left(z;\;\left[\rho \right],T\right)]. \end{array}$$

Although ρ represents a microscopic density field, it is an average quantity; the microscopic degrees of freedom have been integrated out. Accordingly, obtaining the equilibrium structure of the fluid is significantly less computationally demanding than performing the average explicitly with a molecular simulation. Importantly, this efficiency is realized after a one-time training stage: Once the functional has been learned, it can be used to perform thousands of structure and free energy calculations, across many different thermodynamic state points, at minimal additional cost. Furthermore, with neural cDFT, $F_{\text{intr}}^{\text{(ex)}}$ can be obtained by functional line integration of $c^{\left(1\right)}$ (*Materials and Methods*), and subsequently used to compute the grand potential $\Omega =\Omega_{V}\left(\left[\rho \right],T\right)$. In contrast, obtaining free energies from simulations is often an involved and costly procedure comprising many intermediate steps (e.g., thermodynamic integration). To give a sense of the computational gains, in what follows, each calculation with neural cDFT that we present takes on the order of a minute on a standard CPU/GPU; in about one hour wall-clock time on a single GPU (*Materials and Methods*) we can predict ρ(z)—with full microscopic resolution—over a ∼200 nm extent (Fig. 1 and *SI Appendix*, Fig. S2). Where direct comparison to molecular simulations is possible, we also show that ab initio neural cDFT is accurate. In this section, we present a selection of results to validate the approach, with a more extensive set of results shown in *SI Appendix*, Figs. S3 and S4.

### Bulk Structure and Thermodynamics.

Beyond its computational efficiency, an appealing feature of cDFT, especially in the context of an ab initio multiscale modeling framework, is its direct access to thermodynamic properties. For example, for a single-component bulk fluid with density $\rho_{\text{b}}$, the pressure can be obtained directly from $c^{\left(1\right)}$ and $F_{\text{intr}}^{\text{(ex)}}$ without the need for any self-consistent calculation

[5] $$\begin{array}{c} P\left(\rho_{\text{b}},T\right)=k_{\text{B}}T\rho_{\text{b}}\left(1-c^{\left(1\right)}\left(\left[\rho_{\text{b}}\right],T\right)\right)-\frac{F_{\text{intr}}^{\text{(ex)}}\left(\left[\rho_{\text{b}}\right],T\right)}{V}. \end{array}$$

In Fig. 2*B*, we show P against $\rho_{\text{b}}$ for water at different T, described by the RPBE-D3 xc functional. Results are shown both for the neural cDFT using Eq. **5**, and directly from molecular simulations. Not only does the neural cDFT faithfully describe the simulation data, but it also exhibits a van der Waals loop at sufficiently low (subcritical) temperatures. This observation is consistent with a previous neural cDFT study for a Lennard–Jones fluid (44), though in contrast, we have included coexistence simulations directly into the training set. Nonetheless, we expect ab initio neural cDFT—here trained on data from relatively small molecular simulations with MLIPs—to describe liquid–vapor phase equilibria. We explore this point further below.

Despite only being trained on planar inhomogeneous density profiles, the bulk structure of the fluid can also be obtained from neural cDFT. Specifically, the bulk structure factor is provided by the Ornstein–Zernike equation,

[6] $$\begin{array}{c} S\left(k;\;\rho_{\text{b}},T\right)=\frac{1}{1-\rho_{\text{b}}{\hat{c}}_{r}^{\left(2\right)}\left(k;\;\left[\rho_{\text{b}}\right],T\right)}, \end{array}$$

where ${\hat{c}}_{r}^{\left(2\right)}$ is the Fourier transform of the radial two-body direct correlation function of the bulk fluid, $c_{r}^{\left(2\right)}$. As mentioned earlier, the latter is obtained by automatic differentiation of $c^{\left(1\right)}$ from neural cDFT, followed by radial projection (43).

Results for S(k) are presented in Fig. 2*B* for carbon dioxide described by the PBE-D3 xc functional, at a supercritical temperature T=360K. Again, we observe good agreement between the ab initio neural cDFT and results obtained directly from molecular simulations. Note that S(k→0) displays nonmonotonic behavior as $\rho_{\text{b}}$ is varied. This observation suggests that supercritical carbon dioxide exhibits a maximum in the isothermal compressibility; we explore such supercritical behavior in more detail later in the article.

### Liquid–Vapor Coexistence.

We now consider liquid–vapor phase equilibria. To construct the binodal in the $\rho_{\text{b}}$–T plane, we follow a procedure analogous to a direct coexistence simulation. Specifically, for a given T, we find a solution to Eq. **4** with $V_{\text{ext}}=0$, corresponding to liquid–vapor coexistence, i.e., those with an interface between the two phases; from the resulting density profile, we then simply read the densities corresponding to the bulk liquid and vapor phases.

In order to stabilize interfacial solutions, we solve the Euler–Lagrange equation subject to the constraint that the overall density ${\overset{¯}{\rho }}_{L}=L^{-1}\int_{0}^{L}\text{d}z\;\rho \left(z\right)$ is fixed, where L is the length of the system domain in z. Whereas in typical cDFT calculations μ is specified as a control variable, here it acts as the Lagrange multiplier that enforces the constraint. For the systems we investigate, this procedure allows us to effectively mimic the canonical ensemble. This approach is similar in spirit to that of ref. 44, which investigated liquid–vapor coexistence of the Lennard–Jones fluid, though our approach to constraining ${\overset{¯}{\rho }}_{L}$ differs.

Results for both water and carbon dioxide are presented in Fig. 2*C*, with L=20 nm. We also show results from direct coexistence simulations. Overall, we observe excellent agreement between the neural cDFT and the molecular simulations. From the computed binodals, we estimate the critical point from the empirical law of rectilinear diameters and critical exponents (62), yielding critical temperature $T_{\text{c}}$ and critical density $\rho_{\text{c}}$.

For water, the neural cDFT predictions agree well with previous simulation studies: TIP4P/2005 yields $T_{\text{c}}$ of 657 K [cf. 640 K from Vega et al. (63)], RPBE-D3 gives 584 K (22), and SCAN gives 695 K (64). For carbon dioxide, we obtain 300 K for TraPPE, 299 K for PBE-D3, 331 K for BLYP-D3, and 343 K for SCAN-rVV10, all consistent with prior molecular simulations (24, 60, 65). Compared to experiment, for both water (647 K) and carbon dioxide (304 K) (66), the empirical force fields give the best agreement. Among the xc functionals investigated, SCAN provides the best agreement for water, and PBE-D3 for carbon dioxide.

### Confined Fluids.

In the case of liquid–vapor coexistence, inhomogeneity arises naturally at the interface between phases. Away from coexistence, external potentials, such as those from confining boundaries, can induce average inhomogeneous structure in the fluid. To demonstrate the ability of ab initio neural cDFT to accurately describe confined fluids, we take $V_{\text{ext}}$ as two 9–3 Lennard–Jones potentials separated by H, fitted to quantum Monte Carlo data for either a single water or carbon dioxide molecule at a graphene surface (67, 68) (*Materials and Methods*).

As seen in Fig. 2*D*, where we show ρ(z) for confined water described by the SCAN xc functional, ab initio neural cDFT is in excellent agreement with results from molecular dynamics simulations. Note that, in these calculations, we solved the Euler–Lagrange equation (Eq. **4**) subject to the constraint that ${\overset{¯}{\rho }}_{L}$ matches the canonical simulations, and the reported values for μ reflect the resulting Lagrange multiplier.

Fig. 2*D* also demonstrates that thermodynamic properties under confinement are well described. In particular, for supercritical carbon dioxide at T=400K, we have computed a measure of the effective pressure—the total force per unit area A exerted by the fluid on the confining walls—from the derivative of the grand potential with respect to separation between the graphene sheets,

[7] $$\begin{array}{c} \begin{array}{cc} \tilde{P} & \equiv -\frac{1}{A}{\left(\frac{\partial \Omega }{\partial H}\right)}_{A,T,\mu }=-\int_{0}^{L}\text{d}z\;\frac{\text{d}V_{\text{wall}}\left(z\right)}{\text{d}z}\rho \left(z\right), \end{array} \end{array}$$

where the right hand side represents a sum rule (69), with $V_{\text{wall}}$ the external potential of a single wall. Following directly from the standard thermodynamic treatment of inhomogeneous fluids (*SI Appendix*), the effective pressure $\tilde{P}=P+\Pi$ comprises the bulk pressure and the disjoining pressure Π, i.e., the additional mechanical contribution from the fluid required to maintain the separation H (70). In order to compare directly against GCMC simulations, results have been obtained with the TraPPE empirical potential. Again, we observe good agreement between the molecular simulations and the neural cDFT. Note that the left and right hand sides of Eq. **7** provide two routes to computing $\tilde{P}$; here we have presented results using the latter, structural, option. Thermodynamic consistency between the two approaches is assessed in *SI Appendix*, Fig. S8; it is overall very good above the critical point, with more pronounced discrepancies observed at subcritical temperatures.

### Training vs. Validation.

We end this section with a brief comment on the extent to which the results presented so far should be regarded as interpolative reconstructions vs. genuine out-of-sample predictions. The simulation data used to train ab initio neural cDFT were obtained from canonical MD simulations across a range of temperatures, either in the presence of random $V_{\text{ext}}\left(z\right)$ or at direct liquid–vapor coexistence. For the binodals shown in Fig. 2*C*, neural cDFT has therefore been exposed to representative configurations from simulation. However, we note that cDFT minimization can be performed at any temperature across the temperature range, demonstrating its ability to interpolate smoothly between discrete training points (*SI Appendix*, Fig. S5).

Importantly, no bulk simulation data—whether structural or thermodynamic (e.g., free energies, pressures, or chemical potentials)—were provided during training. The bulk equations of state and structure factors shown in Fig. 2*B* should therefore be regarded as genuine held-out predictions, validated directly against the MLIP simulations. Similarly, the values of μ reported in Fig. 2*D* are not known from MD simulations and are instead predictions of neural cDFT. While direct validation of these quantities against MLIP simulations is challenging, the excellent structural agreement between ab initio neural cDFT and MD provides strong indirect support. The effective pressure under confinement, also shown in Fig. 2*D*, is likewise not known a priori. The predictive capability of ab initio neural cDFT beyond its training set is perhaps most clearly illustrated by the fact that $c^{\left(1\right)}\left(z;\;\left[\rho \right],T\right)$—an object defined in the grand canonical ensemble—is learned entirely from data generated in canonical MD simulations.

## Liquid–Vapor Coexistence of Nanoconfined Water

Understanding how confinement influences the behavior of fluids has garnered significant recent interest driven, in part, by advances in fabricating nanofluidic channels (71). In the case of water, recent experiments have reported a profound impact of extreme confinement on its dielectric properties (72, 73) and capillary behavior (74, 75). Many groups have also studied nanoconfined water with molecular simulations, both with traditional empirical potentials and MLIPs (28, 76–81). Importantly, not only are molecular simulations used to cast light on experimental findings, they also act as a predictive tool—a prime example is the prediction of new phases under extreme confinement (28).

When dealing with emergent phenomena such as phase behavior, a consistent thermodynamic description is imperative to fully maximize the predictive potential of molecular modeling (82). For typical molecular simulations, however, how to appropriately discuss relevant thermodynamic variables under confinement can be complicated. For example, a common approach for computing pressure under confinement is to use the lateral components of the virial pressure tensor (28, 76–78). For extreme confinement, however, this measure of pressure depends sensitively on the precise definition of the separation, which is, to a certain extent, arbitrary. In contrast, both P (the pressure of the reservoir) and Π (the disjoining pressure) are insensitive to the precise definition of H, and are readily obtainable within a cDFT formalism. We refer the reader to ref. 69, and we also provide a brief overview in *SI Appendix*.

**Fig. 3.**
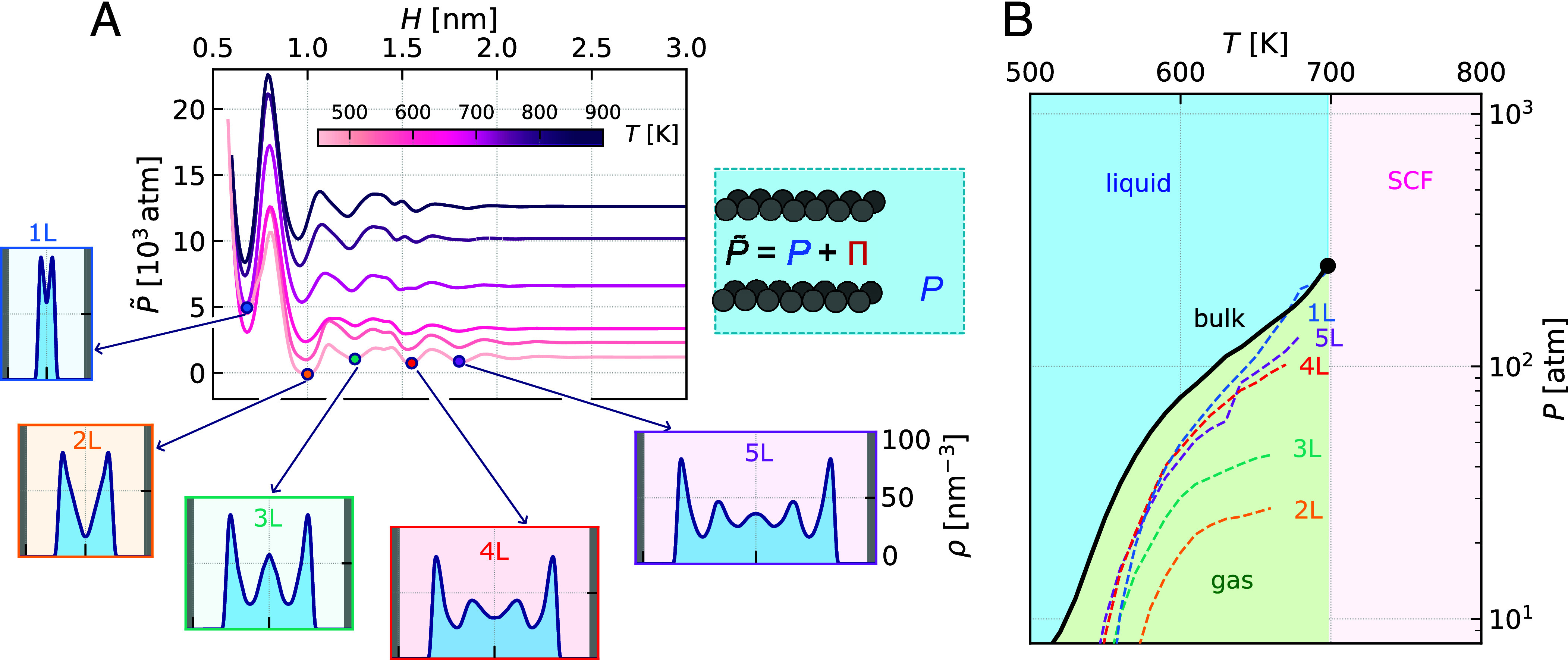
Predicting liquid–vapor equilibria of SCAN water upon confinement between graphene sheets. (*A*) The effective pressure $\tilde{P}$ (Eq. **7**), for a graphene slit pore in equilibrium with a reservoir ($\rho_{\text{b}}=33$ nm^−3^) at different T. $\tilde{P}$ exhibits minima at different H, each corresponding to a different number of water layers, as seen in the accompanying density profiles (the dark shaded regions indicate the positions of the graphene sheets). (*B*) Liquid–vapor phase diagram in the P–T plane, for the different H indicated. Note that P is the bulk pressure of the reservoir.

Our results in Fig. 2*D* already demonstrate the capability of ab initio neural cDFT to describe the structure and thermodynamics of confined fluids. We now capitalize upon both its conceptual and computational advantages to predict, from first principles, how confinement influences water’s liquid–vapor coexistence. In Fig. 3*A*, we present $\tilde{P}$ vs. H for the graphene slit pore in a liquid state in equilibrium with a reservoir at $\rho_{\text{b}}=33$ nm^−3^ at different T. As expected, in all cases we observe $\lim_{H\to \infty }\tilde{P}\to P$. Strikingly, for H≲2 nm, we observe several local minima; these correspond to slit widths that are commensurate with a particular number of layers of water, as can be seen from visual inspection of the density profiles.

While we have shown results for a liquid-like state, under certain conditions, the Euler–Lagrange equation can simultaneously admit a solution corresponding to a vapor-like state; the stable phase is that with lowest Ω. To construct coexistence curves under confinement, we therefore seek, for a given H and T, the value of μ at which the vapor- and liquid-like states have equal grand potentials. With molecular simulation, this procedure—systematically varying μ to locate phase coexistence across a range of H and T—would require either many GCMC simulations, which are impractical with MLIPs (50), or an involved set of free energy calculations. In contrast, with ab initio neural cDFT the entire phase diagram is obtained in minutes. We present the resulting confined liquid–vapor phase diagram in Fig. 3*B*. As the equation of state P(μ,T) is readily obtainable from neural cDFT, to facilitate intuitive understanding, we present results in the P–T plane.

We immediately see from Fig. 3*B* that, relative to bulk, confinement acts to stabilize the liquid phase. In general, we also observe that the critical temperature is shifted down as H decreases, in line with expectations from the standard thermodynamic treatment of inhomogeneous fluids (69). An exception to this trend is the most extreme confinement corresponding to a single layer of water, H≈0.7 nm. As can be deduced from Fig. 3*A*, even though for a given bulk pressure the liquid is still generally stabilized, under this extreme confinement Π>0; this indicates that the fluid exerts a repulsive force on the confining graphene sheets. This effect is made more apparent in *SI Appendix*, Fig. S9, where we show the phase diagram in the $\tilde{P}$–T plane.

## Supercritical Crossover Lines in Carbon Dioxide

So far, we have shown ab initio neural cDFT’s ability to probe liquid–vapor coexistence both in bulk, and under confinement. We now demonstrate its potential to describe the physics of bulk fluids away from coexistence, focusing on supercritical carbon dioxide (scCO2). Understanding scCO2, and supercritical fluids (SCFs) more generally, is not only important from a practical viewpoint—it underpins technologies for carbon capture, sustainable power generation, and chemical extraction (83, 84)—but also of fundamental interest. For example, fluids under extreme conditions are often encountered deep in the interior of giant “gas” planets, and supercritical fluids are known to exhibit nontrivial thermodynamic and dynamic behavior (85, 86).

**Fig. 4.**
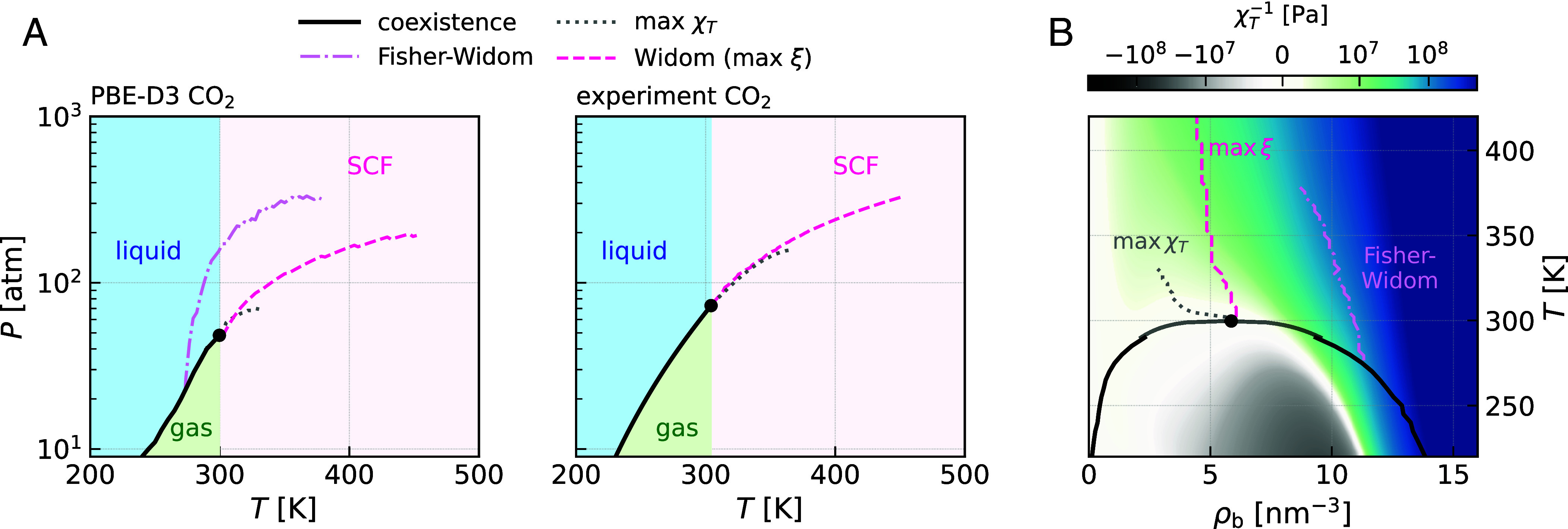
Predicting behavior of supercritical PBE-D3 carbon dioxide. (*A*) P–T phase diagram obtained with ab initio neural cDFT (*Left*) and experiment (*Right*) (87–89); overall, good agreement between the two is observed. (*B*) $\rho_{b}$–T phase diagram with $\chi_{T}^{-1}$ superimposed as a heat map (Eq. **9**). The Widom line obtained by $\max\chi_{T}$ is shown by the dotted line. We also show the Widom line obtained from maxξ, where ξ is the true correlation length (dashed line). The dot-dashed line shows the Fisher–Widom line, indicating a crossover from simple exponential to oscillatory asymptotic decay of the total correlation function. The Widom and Fisher–Widom lines are also plotted in panel (*A*).

Here, we focus on the PBE-D3 xc functional, as we have already established that it reasonably well describes carbon dioxide’s liquid–vapor coexistence, while results for other functionals are shown in *SI Appendix*, Fig. S7. In Fig. 4*A*, we recast the phase diagram in the P–T plane, where we also compare directly to the experimental result. While small differences between theory and experiment are observed, agreement is overall very good. Turning our attention to the supercritical state, we probe the bulk properties of scCO2 by analyzing the total correlation function h(r), whose Fourier transform is related to the two-body direct correlation function of the uniform fluid, via the Ornstein–Zernike equation (12),

[8] $$\begin{array}{c} \hat{h}\left(k\right)=\frac{{\hat{c}}_{r}^{\left(2\right)}\left(k\right)}{1-\rho_{\text{b}}{\hat{c}}_{r}^{\left(2\right)}\left(k\right)}. \end{array}$$

For fluids with short-ranged interatomic potentials, it is well established (90) that, for a given temperature, the asymptotic behavior of h(r) changes from monotonic exponential at low densities, i.e.,

$$\begin{array}{c} h\left(r\right)∼\;\exp\left(-\alpha_{0}r\right), \end{array}$$

to oscillatory with an exponential envelope, i.e.,

$$\begin{array}{c} h\left(r\right)∼\;\cos\left({\tilde{\alpha }}_{1}r-\theta \right)\exp\left(-{\tilde{\alpha }}_{0}r\right). \end{array}$$

The density at which this crossover occurs is the Fisher–Widom (FW) transition (90, 91), and reflects the competition between slowly varying attractive interactions and rapidly varying repulsive forces that govern the packing of molecules; the locus of points in the $\rho_{\text{b}}$–T plane at which this transition occurs is the FW line.

As determining the FW line requires precise characterization of the long-range behavior of h(r), it is extremely challenging to obtain from molecular simulation; accurate determination of the asymptotic decay requires system sizes and sampling times that are computationally prohibitive, particularly for ab initio descriptions. Consequently, previous studies have been limited to simple model fluids (90, 92, 93). With ab initio neural cDFT, the inverse decay lengths, $\alpha_{0}$ and ${\tilde{\alpha }}_{0}$, and the period $2\pi /{\tilde{\alpha }}_{1}$ can be obtained directly from the pole structure of Eq. **8** (*Materials and Methods*); the FW transition is then determined from the density consistent with $\alpha_{0}={\tilde{\alpha }}_{0}$. In Fig. 4*B*, we plot carbon dioxide’s FW line in the $\rho_{\text{b}}$–T plane. In line with previous work for the truncated-and-shifted LJ potential (90), we find that the FW line intersects the binodal at $T/T_{\text{c}}\approx 0.90$ and $\rho_{\text{b}}/\rho_{\text{c}}\approx 1.93$.

A complementary description of the supercritical fluid is offered by the “Widom line” (94–97), which was originally defined by the locus of points in the $\rho_{\text{b}}$–T plane of maximum correlation length, though it is typically associated with maxima in thermodynamic response functions (98–102). As shown in ref. 44 for a LJ fluid, the Widom line can be obtained from the maximum in the true correlation length $\xi =1/\alpha_{0}$, which we show in Fig. 4*B*. We also present the Widom line obtained from the maximum in isothermal compressibility,

[9] $$\begin{array}{c} \chi_{T}\left(\rho_{\text{b}},T\right)=\frac{\beta }{\rho_{\text{b}}}S\left(k=0;\;\rho_{\text{b}},T\right), \end{array}$$

which deviates from the Widom line obtained from maxξ, in a manner similar to that observed for the LJ fluid. Both sets of Widom lines are also plotted on the P–T phase diagrams in Fig. 4*A*. In this case, the discrepancy between the two is less pronounced, with the Widom line computed from $\max\chi_{T}$ lying slightly below that obtained from maxξ.

## Discussion

In this work, we have introduced ab initio neural cDFT as a simple, first-principles multiscale modeling framework for fluids. A distinctive aspect of ab initio neural cDFT compared to typical multiscale methods is that it treats physics across length scales within the same theoretical framework. In addition to obtaining the equilibrium structure of the fluid, a key appealing feature of the approach is its treatment of thermodynamics, for both homogeneous and inhomogeneous systems. In particular, as neural cDFT is formulated in the grand canonical ensemble, the chemical potential of the fluid is known, and measures of pressure in confined systems are well defined and readily obtainable. We capitalized upon this feature to obtain the liquid–vapor coexistence curves of water confined between graphene sheets, with interatomic interactions described by the SCAN xc functional. We also obtained the Fisher–Widom and Widom lines in supercritical carbon dioxide, with interatomic interactions described by the PBE-D3 xc functional; this demonstrates the framework’s ability to even describe bulk properties that would be challenging to obtain from molecular simulations alone.

It is important to stress that our aim has been to introduce a framework that provides accurate predictions of a fluid’s emergent physics equipped only with knowledge of its intermolecular interactions, as determined by the Schrödinger equation; we have not made any attempt to improve the necessary approximations to the many-body electronic structure problem. In this spirit, to the extent that the MLIPs provide suitable surrogate models, comparison to experimental data should be viewed as informing on the appropriateness of the underlying interatomic potential for the problem at hand, rather than an assessment of the multiscale framework itself. Whether ab initio neural cDFT can be useful for improving or validating the underlying electronic structure approximation, e.g., by extending validation metrics to more mesoscopic properties, remains an interesting question that lies beyond the scope of the current study.

In the same vein, we have also used MLIPs that are readily available (22, 24, 64), rather than trying to improve their description of the potential energy surface; we have neither generated additional training data, nor developed a new MLIP architecture. This means we have used MLIPs that rely on an atom’s local environment to determine the force acting upon it; explicit electrostatic interactions are missing. Such neglect of long-ranged interactions can have important consequences for dielectric fluids such as water. In bulk, simulations reveal that the subsequent lack of screening causes long wavelength polarization fluctuations that differ from expected behavior, while for interfacial systems, erroneous polarization gradients can be observed (103–107); a recent study of water at a copper substrate has even found that short-ranged MLIPs can lead to false metallization of the liquid layer (108).

Even for carbon dioxide, we cannot rule out the possibility that the Fisher–Widom line reported in Fig. 4 is a consequence of the local description provided by the MLIP; the asymptotic behavior of correlations may be influenced by both interactions between permanent quadrupoles and London dispersion forces (109, 110). Even if this is the case, the results discussed here likely provide a useful starting point for understanding the physics of the supercritical state. We also emphasize that our aim is to demonstrate how ab initio neural cDFT can be used to assess the statistical mechanics of the underlying intermolecular potential in ways that can be challenging for molecular simulations. Clearly, a completely rigorous description ought to take the effects of long-ranged electrostatics explicitly into account, and we note recent progress from several groups in this regard (106, 107, 111–119).

Yet, by and large, for the properties that we have investigated, it is likely that this neglect of long-ranged correlations is not too severe. In particular, previous studies comparing long-ranged and short-ranged MLIPs have found that liquid–vapor density profiles are virtually indistinguishable (107, 112). In a careful analysis, using ideas from local molecular field theory (LMFT) (104, 105, 120, 121), Niblett et al. showed that short-ranged MLIPs attempt to learn the effects of long-ranged interactions implicitly, albeit in an uncontrolled manner. (112). With the assumption that $c^{\left(1\right)}$ only depends on nearby values of the density, locality also underpins the machine-learning approach used in neural cDFT; it is this locality that enables predictions on length scales far beyond the reach of molecular simulations (Fig. 1 and *SI Appendix*, Fig. S2) (43). Recently, building on ideas from LMFT, we have shown how the effects of long-ranged electrostatics can be accurately accounted for using a well-controlled mean-field approximation (47). With the ability to model inhomogeneous systems over large length scales—where subtle effects of long-ranged correlations are likely to become pronounced—neural cDFT potentially provides a means to test MLIPs in ways that would be challenging with molecular simulations alone.

For molecular liquids, accounting for electrostatic interactions with neural cDFT relies on hyper-DFT, an extension of traditional cDFT that permits the description of equilibrium observables (49, 122); in refs. 48 and 49, we treated the charge density as the observable. But hyper-DFT can be employed to access other observables that might be of interest. For example, in *SI Appendix*, Fig. S10, we show how the density profile of confined water’s hydrogen atoms can be obtained, in addition to the one-body density prescribed by the oxygen atoms. Importantly, the hyper-DFT framework ensures that ab initio neural cDFT can readily accommodate MLIPs that explicitly treat long-ranged electrostatics (49), and more generally, offers a route to formulating theoretical descriptions of fluid behavior. For example, we recently used hyper-DFT to investigate how electric field gradients influence the capillarity of dielectric fluids (48).

As we acknowledged earlier, aside from the structure of the bulk fluid, the neural cDFT we trained is limited to describing systems with planar inhomogeneous geometries. For the confined systems we have studied, this limitation means that the external potential approximates the effective interaction between the substrate and the fluid. Previous simulation studies that have directly compared such implicit substrate potentials against explicit atom representations suggest that many of the most salient aspects of confinement are well described (28, 123). Even in cases where an explicit atom representation of the substrate is important, ab initio neural cDFT may prove useful in providing a reasonable estimate of the system’s thermodynamic state. Ab initio neural cDFT is also limited to describing the structure and thermodynamics of fluids at equilibrium. For overdamped dynamics, it is possible to apply a similar machine-learning approach to nonequilibrium scenarios (124), but the applicability to molecular systems is questionable. Even with these limitations in mind, the results we present make clear that ab initio neural cDFT is in a position to cast light onto the influence of complex microscopic interactions on emergent properties of fluids, and represents a significant advancement in the ab initio multiscale modeling of liquids.

Looking forward, one can envisage extending our strategy into a fully machine-learned hierarchy spanning multiple physical scales. Recent advances in machine-learned xc functionals (125–127) could provide electronic structure data from which MLIPs are derived, which in turn generate the data used to construct neural cDFT. Such a pipeline would constitute a unified, fully machine-learned multiscale framework linking electronic structure to the emergent physics of liquids. In the meantime, the integration of MLIPs and neural cDFT provides a route to multiscale fluid modeling in which both the thermodynamics of open systems and mesoscale structure become readily accessible, while maintaining a direct connection to the underlying first-principles Hamiltonian.

## Materials and Methods

### Machine-Learned Interatomic Potentials.

For carbon dioxide, the training datasets for energies and forces were taken from ref. 24. We considered three xc functionals: PBE (57), BLYP (58) with D3 dispersion corrections (55), and SCAN-rVV10 (53, 59). For each xc functional we trained an MLIP using the DeepMD architecture with the DeePMD-kit package (128), minimizing the loss function

$$\begin{array}{c} \begin{array}{cc} L & =p_{E}\sum_{k=1}^{n}{\left|E_{\text{ref}}\left(R_{k}^{N}\right)-E\left(R_{k}^{N}\right)\right|}^{2}\\ & +p_{f}\sum_{k=1}^{n}\sum_{i=1}^{N}{\left|\left|f_{i,\text{ref}}\left(R_{k}^{N}\right)-f_{i}\left(R_{k}^{N}\right)\right|\right|}^{2}, \end{array} \end{array}$$

where $p_{E}$ and $p_{f}$ are tunable parameters that varied during the optimization, $R_{k}^{N}$ is the $k^{\mathrm{th}}$ atomic configuration in the training set with atomic forces $f_{i,\text{ref}}\left(R_{k}^{N}\right)$ and energy $E_{\text{ref}}\left(R_{k}^{N}\right)$, while $f_{i}\left(R_{k}^{N}\right)$ and energy $E(R_{k}^{N})$ are the corresponding model predictions (i=1…N indexes the atoms). For reasons of computational efficiency, we employed DeepMD for the carbon dioxide MLIPs used in the main article. However, we also trained an MLIP for PBE-D3 carbon dioxide using MACE to show the robustness of the method to different MLIP architectures (*SI Appendix*, Fig. S1). For water, we considered two xc functionals: RPBE (54) with D3 dispersion corrections, and SCAN. For RPBE-D3 water, we used the MLIP trained in ref. 22 based upon the HD-NNP architecture. For SCAN water, we used the MLIP trained in ref. 64 based upon the DeepMD architecture.

### Generation of Training Data for Neural cDFT.

To obtain training data for the neural network representations of $c^{\left(1\right)}\left(z;\;\left[\rho \right],T\right)$, for each fluid and intermolecular potential we performed MD simulations under random $V_{\text{ext}}$ of the form (43)

$$V_{\text{ext}}\left(z\right)=\sum_{n=1}^{4}A_{n}\sin\left(\frac{2\pi nz}{L_{z}}+\theta_{n}\right)+\sum_{n=1}^{4}B_{n}^{\text{lin}}\left(z\right),$$

where $L_{z}$ is the simulation box length in the z direction. The phases $\theta_{n}$ were chosen uniformly in the interval [0, 2π), and values of $A_{n}$ were drawn from an unbiased normal distribution with variance of $0.4\;{\left(k_{\text{B}}T\right)}^{2}$. The second summation denotes up to four piecewise linear functions

$$B_{n}^{\text{lin}}\left(z\right)=\left\{\begin{array}{cc} V_{1}+\frac{V_{2}-V_{1}}{z_{2}-z_{1}}\left(z-z_{1}\right) & z_{1}<z<z_{2}\\ 0 & \text{otherwise}, \end{array}\right.$$

with $0<z_{1}<z_{2}<L_{z}$. The locations $z_{1}$ and $z_{2}$ were distributed uniformly while $V_{1}$ and $V_{2}$ were chosen randomly from an unbiased normal distribution with variance of $0.8\;{\left(k_{\text{B}}T\right)}^{2}$. From these randomized $V_{\text{ext}}$, the external force $f_{\text{ext}}\left(z\right)=-\partial_{z}V_{\text{ext}}\left(z\right)$ was obtained by finite difference and added to the molecular centers (i.e., the carbon atom for carbon dioxide, and the oxygen atom for water). In some cases, planar walls of the form of a 9–3 Lennard–Jones potential,

$$V_{\text{wall}}\left(z\right)=\epsilon_{\text{wall}}\left[\frac{2}{15}(\frac{\sigma_{\text{wall}}}{z}{)}^{9}-(\frac{\sigma_{\text{wall}}}{z}{)}^{3}\right]$$

were also included, with $\epsilon_{\text{wall}}\in$ [0.02, 2]$k_{\text{B}}T$ and $\sigma_{\text{wall}}\in \left[0.1,0.3\right]\;$nm.

Simulation cells of size $3.332\times 3.332\times 3.332\;{\text{nm}}^{3}$ for carbon dioxide and $2.000\times 2.000\times 4.000\;{\text{nm}}^{3}$ for water were used, and periodic boundary conditions were employed. MD simulations were performed in the canonical ensemble, from which the density profiles were sampled after at least 1 ns of production run. The number of molecules in the box was randomized between 70 and 512 for carbon dioxide and 40–1,024 for water. For TraPPE carbon dioxide, the linear molecules are evolved as rigid bodies (129). For TIP4P/2005 water, the geometries of the water molecules were constrained using the RATTLE algorithm (130). Dynamics were propagated using the velocity Verlet algorithm with a time-step of 0.5 fs. The temperature, chosen randomly between 220 and 500 K for carbon dioxide and 450 to 950 K for water, was maintained using a Nosé–Hoover thermostat (131, 132). We used the LAMMPS simulation package (133) interfaced with the n2p2 package (134) for the HD-NNP potential and the DeepMD-kit package for the DeepMD potentials. Overall, for each fluid and interatomic potential, we sampled 500 to 1,000 simulations to generate the training data for neural cDFT.

We also performed direct coexistence MD simulations with simulation cell sizes $3.332\times 3.332\times 20.000\;{\text{nm}}^{3}$ for carbon dioxide (comprising 1,536 molecules) and $2.000\times 2.000\times 20.000\;{\text{nm}}^{3}$ for water (comprising 832 molecules). For each interatomic potential, approximately 20 simulations were performed at random T from 220 K up to $T_{\text{c}}$ for carbon dioxide, and from 450 K up to $T_{\text{c}}$ for water. Density profiles were obtained after at least 1 ns production run.

### Training Neural cDFT.

To learn $c^{\left(1\right)}\left(\left[\rho \right],T\right)$ for each fluid and interatomic potential, we employed a local learning strategy detailed elsewhere (43, 51). In brief, inputs consisted of local density in a sliding spatial window of size 1nm from the center of the position of interest, along with a separate input node that encodes T as a scalar. The loss function is defined based on Eq. **4** as

$$L=\sum_{k=1}^{n}{\left|\left|\ln(\zeta^{-1}\Lambda^{3}\rho_{k}\left(z\right))+\beta_{k}V_{\text{ext}}^{\left(k\right)}\left(z\right)-\beta_{k}\mu_{k}-c^{\left(1\right)}\left(z;\;\left[\rho_{k}\right],T_{k}\right)\right|\right|}^{2},$$

where k indexes each simulation in the dataset. Note that, as we have used canonical MD simulations to generate our training data, $\left\{\mu_{k}\right\}$ are treated as latent variables that are learned during the training procedure. In practice, we set $\zeta^{-1}\Lambda^{3}=1\;$Å^3^.

The machine-learning routine was implemented in Keras/Tensorflow (135). One fifth of the dataset was used for validation, and the rest was used for training. Models were trained for 100 epochs with a batch size of 128, using an exponentially decaying learning rate starting at 0.001, achieving errors comparable to the estimated simulation noise. The network contains three fully connected layers, each containing 128, 64, and 32 nodes respectively, with softplus activation. The training of the neural networks was done on a GPU (NVIDIA GeForce RTX 3060) in a few hours.

### Evaluating Neural cDFT.

Evaluating the trained neural functionals is fast (∼milliseconds) and can be performed on a CPU or GPU. The EL equation was solved self-consistently with a mixed Picard iteration scheme, which typically converges within minutes. When constraining ${\overset{¯}{\rho }}_{L}$, μ acts as the Lagrange multiplier, which is achieved by a primal–dual gradient scheme.

To evaluate the excess free energy, we use functional line integration,

$$\beta F_{\text{intr}}^{\text{(ex)}}\left(\left[\rho \right],T\right)/A=-\int_{0}^{1}\text{d}\lambda \int \text{d}z\;\rho \left(z\right)\;c^{\left(1\right)}\left(z;\;\left[\lambda \rho \right],T\right),$$

where A is the cross-sectional area.

To obtain the Widom and Fisher–Widom lines, a pole analysis was performed following refs. 44 and 90, i.e., by finding the zeros of the denominator $1-\rho {\hat{c}}_{r}^{\left(2\right)}\left(\alpha \right)$ in Eq. **6**. Here, $\alpha \left(\rho_{\text{b}},T\right)=\alpha_{1}+i\alpha_{0}$, with $\alpha_{0}$ and $\alpha_{1}$ satisfying

$$1=4\pi \rho_{\text{b}}\int_{0}^{\infty }\text{d}r\;r^{2}c_{r}^{\left(2\right)}\left(r;\;\left[\rho_{\text{b}}\right],T\right)\frac{\sinh(\alpha_{0}r)}{\alpha_{0}r}\cos\left(\alpha_{1}r\right),$$

$$1=4\pi \rho_{\text{b}}\int_{0}^{\infty }\text{d}r\;r^{2}c_{r}^{\left(2\right)}\left(r;\;\left[\rho_{\text{b}}\right],T\right)\cosh\left(\alpha_{0}r\right)\frac{\sin(\alpha_{1}r)}{\alpha_{1}r}.$$

### Validation Simulations.

For the simulation data in Fig. 2, to obtain the pressure and structure factors, we performed simulations of the bulk fluid with simulation cells $3.332\times 3.332\times 3.332\;{\text{nm}}^{3}$ for carbon dioxide, and $2.000\times 2.000\times 2.000\;{\text{nm}}^{3}$ for water. Direct coexistence simulations were performed as described above (*Generation of Training Data for Neural cDFT*). For simulations of the confined fluids, we employed Lennard–Jones 9–3 walls (with a 1 nm cutoff), separated by H. To avoid interactions between periodic images, the box dimension $L_{z}$ was chosen such that $L_{z}-H=4\;\text{nm}$. The wall–fluid interaction parameters were obtained by fitting to quantum Monte Carlo data (67, 68), yielding $\epsilon_{\text{wall}}=0.1298\;\text{eV}$, $\sigma_{\text{wall}}=0.3868\;\text{nm}$ for carbon dioxide and $\epsilon_{\text{wall}}=0.0829\;\text{eV}$, $\sigma_{\text{wall}}=0.3590\;\text{nm}$ for water. When benchmarking the disjoining pressure of TraPPE carbon dioxide, we performed GCMC simulations with our own code (136).

## Supplementary Material

Appendix 01 (PDF)

## Data Availability

The code used to train the models and perform neural cDFT calculations in this study is available on Github https://github.com/annatbui/mlip-neuraldft (137). Example simulation inputs, the MLIP models, and the training data are available on Zenodo https://zenodo.org/records/21245362 (138).
