# Supplementary material for: A unified machine-learning framework for ab initio multiscale modeling of liquids
Source: Proc Natl Acad Sci U S A. 2026 Jul 24;123(30):e2610049123. doi: 10.1073/pnas.2610049123 (PMC13416084; doi:10.1073/pnas.2610049123)
Supplement: Supplementary file 1 — Appendix 01 (PDF) [file pnas.2610049123.sapp.pdf]

Supporting Information for

A unified machine learning framework for *ab initio* multiscale modeling of liquids

Anna T Bui and Stephen J Cox

Stephen J Cox.
E-mail: stephen.j.cox@durham.ac.uk

This PDF file includes:

Supporting text
Figs. S1 to S10
Table S1
SI References

Supporting Information Text

Applicability to different MLIP architectures

To generate the training data for the neural cDFT, we have employed MLIPs with the DeepMD (1) architecture for carbon dioxide with all xc functionals considered (PBE-D3, BLYP, SCAN-rVV10). We also used DeepMD for SCAN water. For RPBE-D3 water, we employed the HD-NNP (2) MLIP architecture. To gauge whether training with the random V_{ext} is sensitive to the choice of MLIP architecture, we also trained an MLIP employing MACE (3) for PBE-D3 carbon dioxide, using the same dataset from Ref. (4) as for the DeepMD MLIP. As seen in Fig. S1, results obtained with MACE are virtually indistinguishable from those with DeepMD, suggesting that the generation of training data for neural cDFT is robust to any reasonable choice of MLIP architecture.

Multiscale prediction

The local nature of the trained neural functionals means that they are able to make efficient predictions on mesoscopic length scales. As an example, in Fig. S2 we present results for PBE-D3 carbon dioxide in which $\beta V_{\mathrm{ext}}(z)$ decreases linearly over 200 nm, along with 25 regions, each with a 1 eV bias of thickness 0.5 nm, separated from each other by 2 nm. It can clearly be seen that *ab initio* neural cDFT simultaneously describes the large length scale redistribution of the fluid and the fine microscopic structure. These results were obtained in approximately one hour on a GPU.

Additional results across interatomic potentials

Using neural cDFT, we show predictions across all interatomic potentials investigated for the equation of state, the structure factor, and fluid structure under confinement. Results for water are shown in Fig. S3, and those for carbon dioxide in Fig. S4. These correspond to genuine out-of-sample predictions, as equations of state and structure factors from MD simulations are not included in the training. The cDFT predictions for liquid–vapor binodals are shown in Fig. S5, demonstrating smooth interpolation of coexistence properties between discrete training temperatures. Corresponding interfacial density profiles are shown in Fig. S6. To quantify these results, Table S1 reports root-mean-square errors (RMSEs) between cDFT predictions and simulation data. In the main text, Fig. 4 shows results for PBE-D3 carbon dioxide; in Fig. S7 we present the corresponding results for the other interatomic potentials.

Thermodynamics of confined fluids

In the main article, we consider single-component fluids confined between two graphene sheets separated by H. In the standard thermodynamic treatment of confined fluids (5, 6), the exact differential of the grand potential is

$$\mathrm{d}\Omega = -S\mathrm{d}T - P\mathrm{d}V - N\mathrm{d}\mu + 2\gamma\mathrm{d}A - \Pi A\mathrm{d}H', \quad [1]$$

where S is the entropy, Π is the disjoining pressure, P is the bulk pressure of the reservoir, and γ is the substrate–fluid interfacial tension. Note that H' is defined by a reasonable choice of dividing surface; in general $H' \neq H$. As $V = AH'$, it immediately follows that

$$-\frac{1}{A}\left(\frac{\partial\Omega}{\partial H'}\right)_{A,T,\mu} = P + \Pi, \quad [2]$$

which we define as the effective pressure $\tilde{P}$. Even though in general $H' \neq H$, $\tilde{P}$ is insensitive to the precise choice of H'. This can be seen immediately by writing $H' = H + \delta H$, such that $\mathrm{d}H' = \mathrm{d}H$. For this reason, we adopt the simple choice $H' = H$.

Assessing thermodynamic consistency

As discussed in the main article, there are two routes to calculating $\tilde{P}$. The thermodynamic route is

$$\tilde{P} = -\frac{1}{A}\left(\frac{\partial\Omega}{\partial H}\right)_{A,T,\mu}, \quad [3]$$

and the structural route is

$$\tilde{P} = -\int_0^L \mathrm{d}z\, \rho(z)\frac{\mathrm{d}V_{\mathrm{wall}}(z)}{\mathrm{d}z}. \quad [4]$$

That is, we can either calculate the derivative of the grand potential, or we can integrate $\rho(z)(\mathrm{d}V_{\mathrm{wall}}/\mathrm{d}z)$. In principle, both approaches should give the same result. In Fig. S8A we show Ω vs. H for TraPPE carbon dioxide confined between two graphene sheets. In Fig. S8B, we compare the resulting $\tilde{P}$ to that presented in the main article, which were obtained by the structural route. At supercritical temperatures, we see that thermodynamic consistency is overall very good. At subcritical temperatures, more pronounced discrepancies are observed; this is due to an accumulation of numerical errors when integrating through intermediate densities in the van der Waals loop (7).

Phase diagram

In addition to the P–T phase diagram of confined water presented in Fig. 3 in the main paper, where P is the bulk pressure of the reservoir, neural cDFT also allows construction of the $\tilde{P}$–T phase diagram, where $\tilde{P} = P + \Pi$. We show this corresponding phase diagram in Fig. S9.

Hyper-DFT to obtain other equilibrium observables

While cDFT provides structural information based on the one-body density ρ, hyper-DFT (8) is a recent extension that states that any equilibrium observable of the fluid can be written as a functional of the one-body density (the "hyperdensity functional"). Here, as an example, we consider the density of the hydrogen atoms ρ_{H} in water as the observable of interest; recall that we use the oxygen position to define the one-body density ρ. For an inhomogeneous fluid, once the equilibrium density $\rho(z)$ is determined from solving the Euler–Lagrange equation, the equilibrium hydrogen density can be obtained by evaluating

$$\rho_{\mathrm{H}}(z) = \rho_{\mathrm{H}}^{(1)}(z; [\rho], T), \qquad [5]$$

where $\rho_{\mathrm{H}}^{(1)}(z; [\rho], T)$ is the corresponding hyperdensity functional.

To obtain a neural functional representation of $\rho_{\mathrm{H}}^{(1)}(z; [\rho], T)$, for each training simulation, we sample not only the equilibrium one-body density $\rho(z)$ centered on the oxygen atoms but also the equilibrium hydrogen density $\rho_{\mathrm{H}}(z)$. Then the mapping $\{\rho(z), T\} \to \rho_{\mathrm{H}}(z)$ is learned locally with a neural network, analogous to the training of $c^{(1)}(z; [\rho], T)$ (see *Methods* section in the main paper). The prediction of the hydrogen density for confined water described with the SCAN xc functional is shown in Fig. S10.

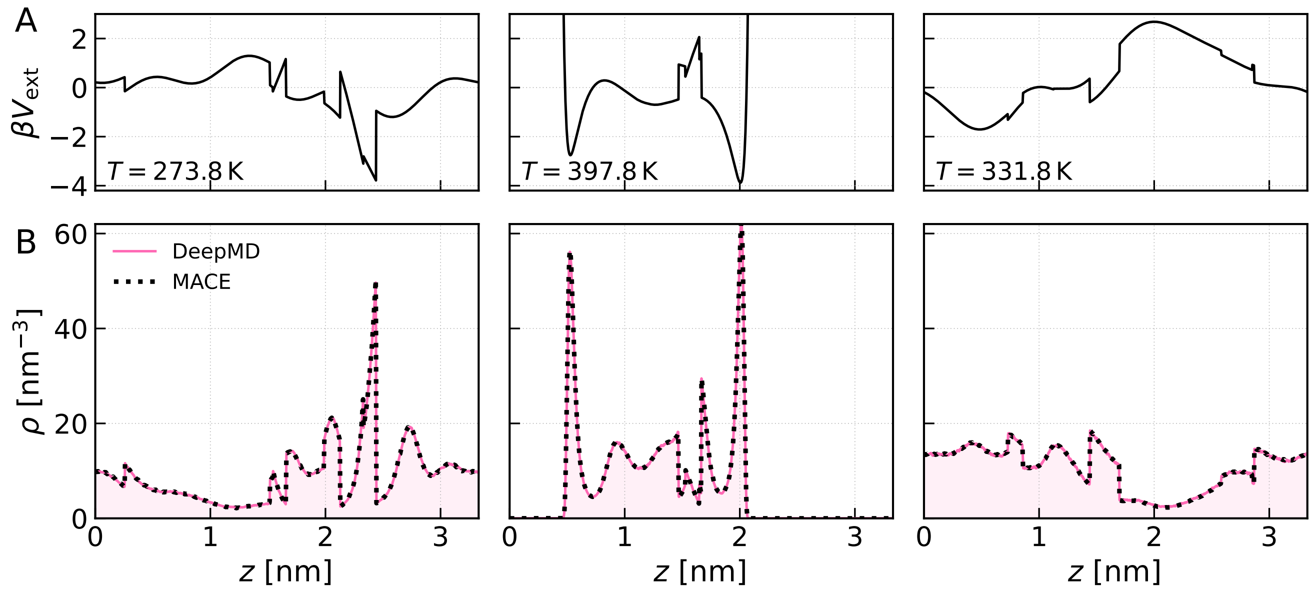

Fig. S1. Robustness to choice of MLIP architecture. For random external potentials applied (A), the density profiles obtained from MD simulation (B) of PBE-D3 carbon dioxide are insensitive to the choice of DeepMD or MACE.

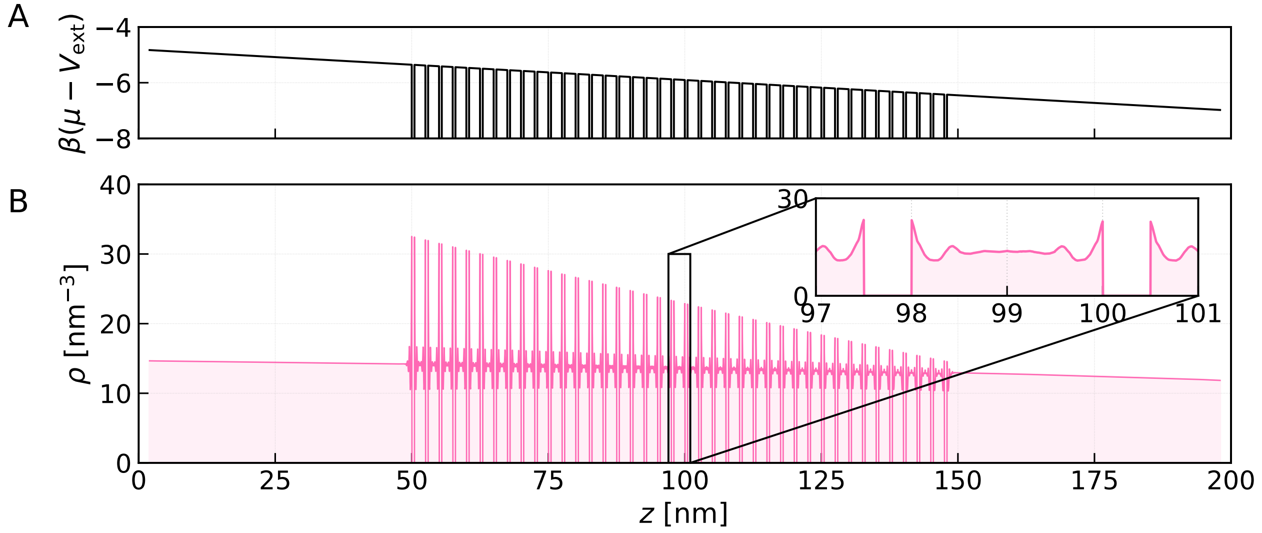

Fig. S2. Mesoscale prediction of PBE-D3 carbon dioxide. For carbon dioxide at $T = 300$ K distributed across a multilayered membrane, with a concentration gradient modeled through a linear bias component in the external potential in A, the density profile is shown in B.

Table S1. Quantitative errors of cDFT predictions against simulations. Root-mean-square errors (RMSE) are reported for bulk pressure P, structure factor $S(k)$, density profiles $\rho(z)$, and coexistence densities $\{\rho_\mathrm{v}, \rho_\mathrm{l}\}$. For each observable, the RMSE is computed over all overlapping data points for cDFT and simulations in the relevant variable (density, wavevector, spatial coordinate, or temperature).

	Water			Carbon dioxide			
RMSE	RPBE-D3	SCAN	TIP4P/2005	PBE-D3	SCAN-rVV10	BLYP-D3	TraPPE
P [10^3 atm]	0.33	0.79	0.21	0.13	-	-	0.01
$S(k)$	0.033	0.033	0.024	0.044	-	-	0.018
$\rho(z)$ [nm^{-3}]	0.64	0.58	0.24	0.86	1.28	0.69	0.20
ρ_v [nm^{-3}]	0.33	0.26	0.16	0.15	0.17	0.04	0.05
ρ_l [nm^{-3}]	0.22	0.12	0.49	0.11	0.15	0.10	0.08

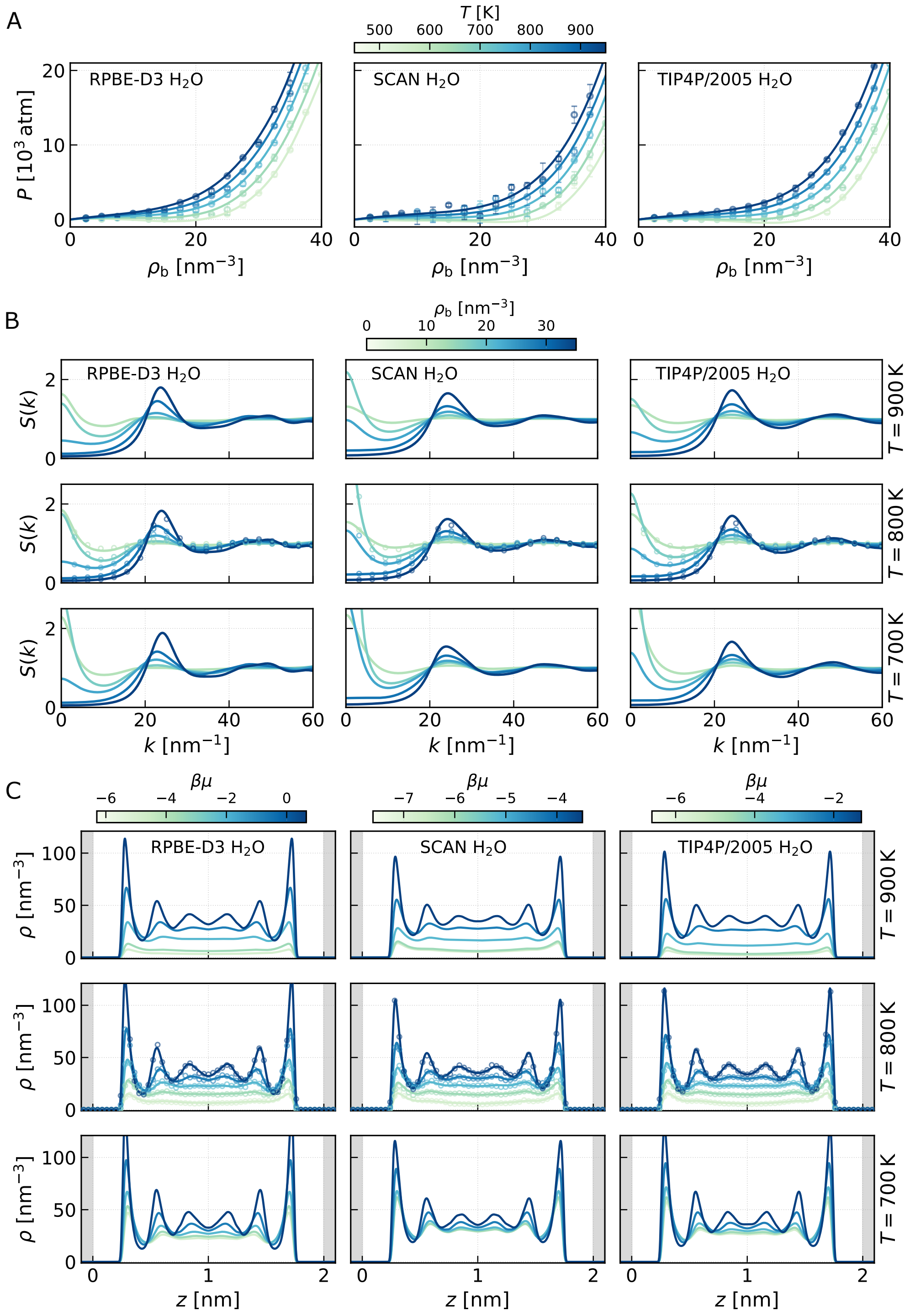

Fig. S3. ***Ab initio*** **structure and thermodynamics of water.** Neural cDFT prediction for water described with the xc functionals RPBE-D3 and SCAN, and the TIP4P/2005 classical force field are shown with solid lines. A: The equation of state. B: The structure factor. C: The structure of the fluid confined between two graphene sheets. Where atomistic simulations have also been performed, we show the corresponding results with symbols.

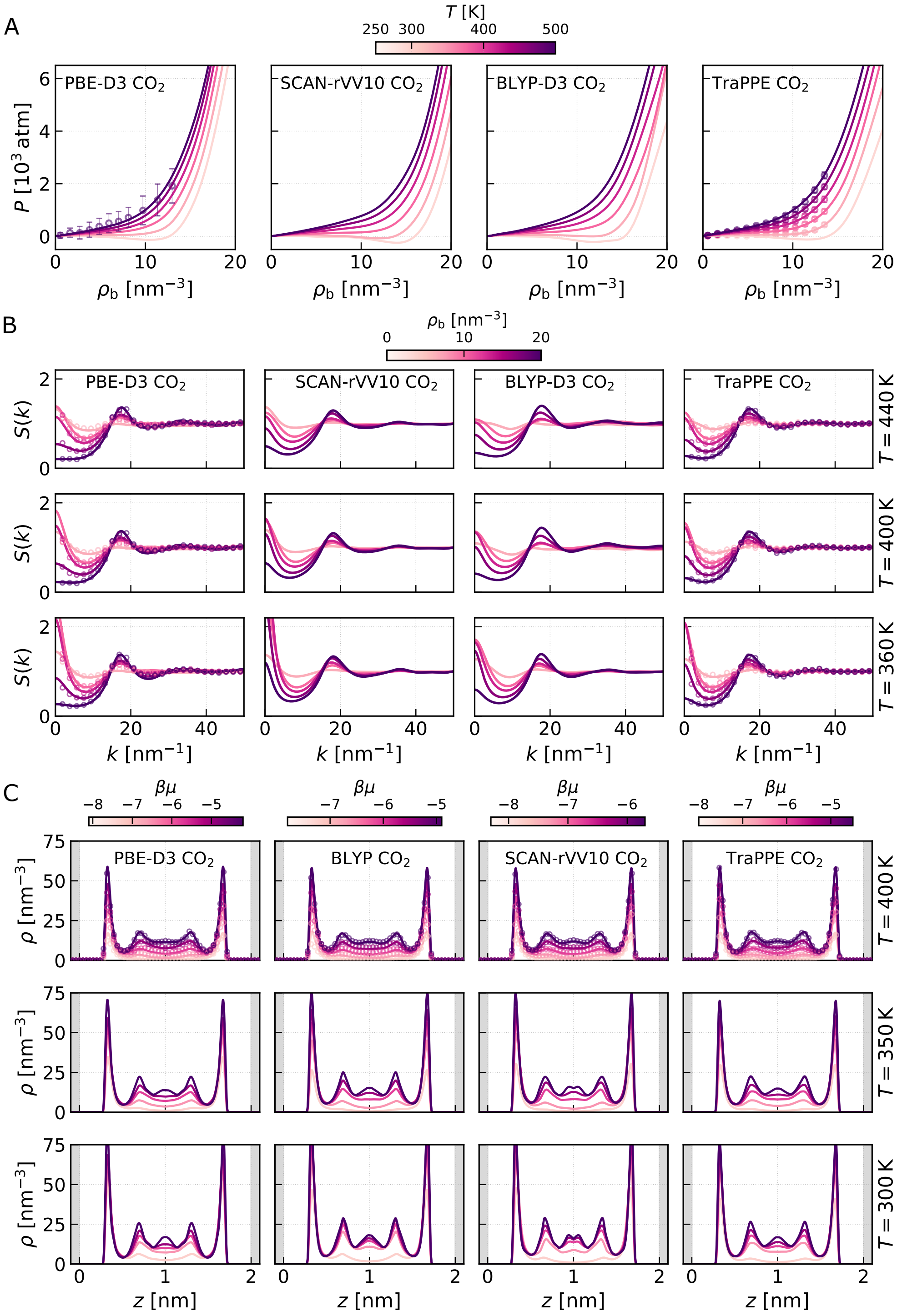

Fig. S4. ***Ab initio*** **structure and thermodynamics of carbon dioxide.** Neural cDFT prediction for carbon dioxide described with the xc functionals PBE-D3, SCAN-rVV10 and BLYP-D3, and the TraPPE classical force field are shown with solid lines. A: The equation of state. B: The structure factor. C: The structure of the fluid confined between two graphene sheets. Where atomistic simulations have also been performed, we show the corresponding results with symbols.

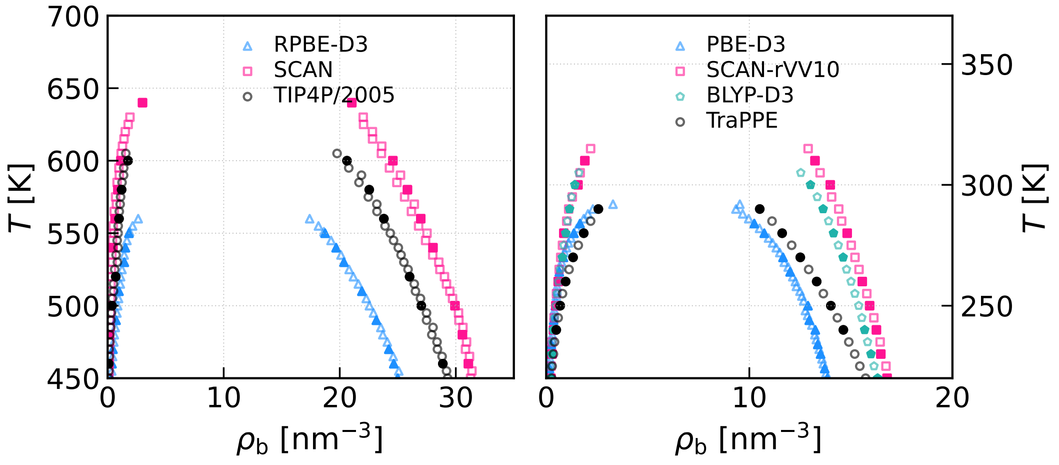

Fig. S5. cDFT prediction of liquid–vapor binodals. Open markers indicate temperatures not included in the training set, while filled markers correspond to temperatures included in the training. These results demonstrate smooth interpolation across thermodynamic conditions.

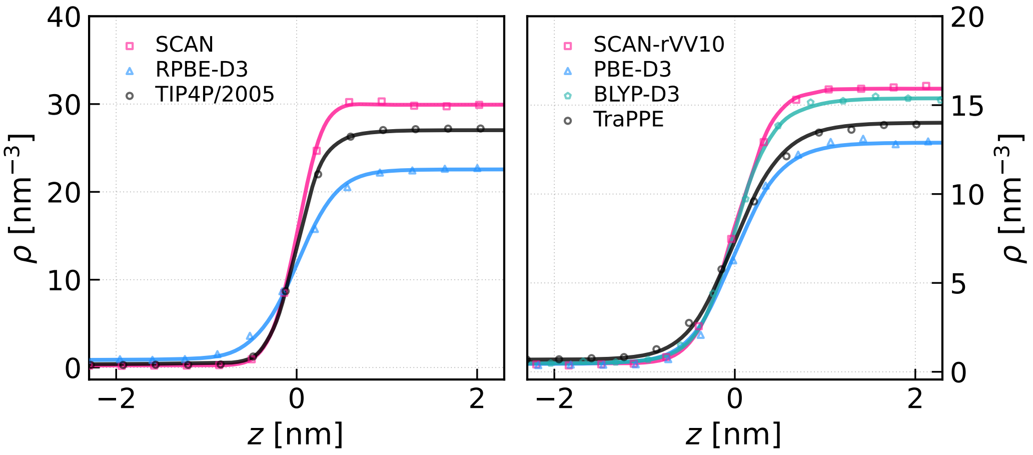

Fig. S6. The liquid-vapor interface. Density profiles $\rho(z)$ at coexistence for water at $T = 500$ K (left) and carbon dioxide at $T = 250$ K (right), corresponding to Fig. 2B of the main text. Profiles are aligned by the Gibbs dividing surface, approximated by the point at which $\rho(z)$ is equal to half the density of the liquid phase.

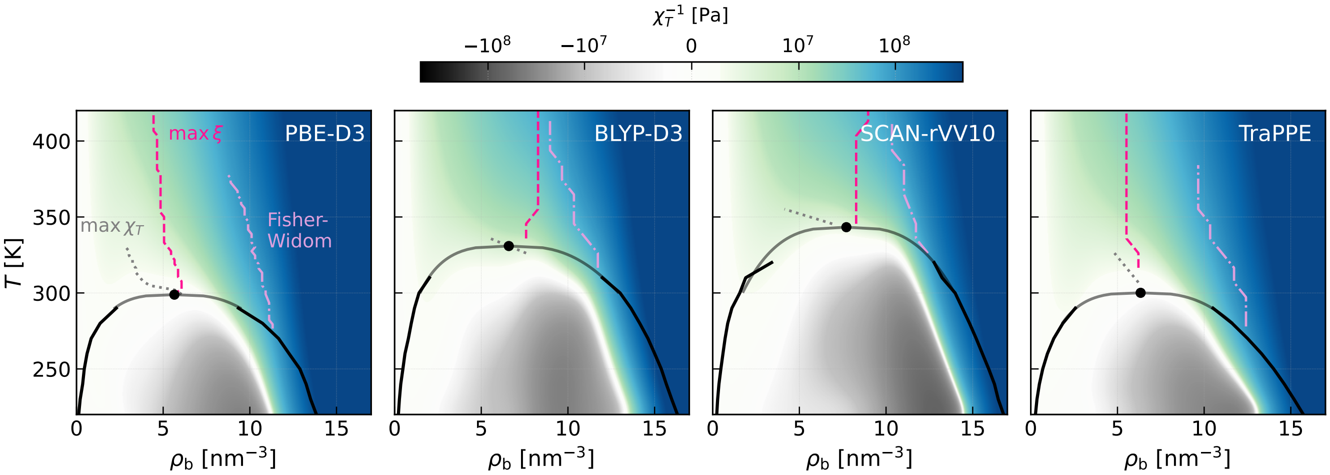

Fig. S7. Supercritical carbon dioxide across different interatomic potentials. ρ_b–T phase diagram with χ_T^{-1} shown as a heat map. The Widom line obtained from $\max \chi_T$ is shown as a dotted line. The dashed line shows the Widom line from $\max \xi$, where ξ is the correlation length. The dot-dashed line indicates the Fisher–Widom line, marking the crossover from monotonic to oscillatory decay of the total correlation function.

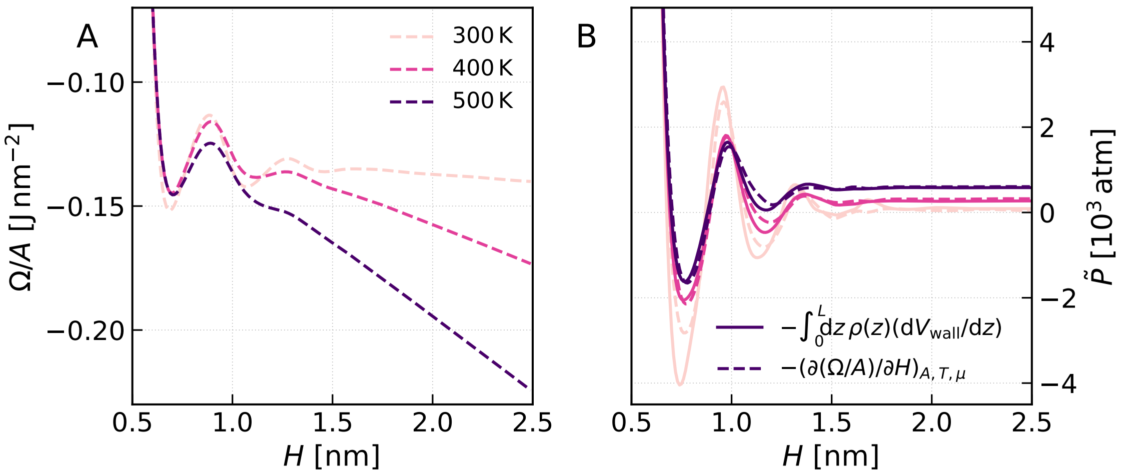

Fig. S8. Assessing thermodynamic consistency in neural cDFT. A: Ω vs. H for TraPPE carbon dioxide confined between two graphene sheets. B: Resulting $\tilde{P}$ compared to results in the main article, which were obtained by the structural route.

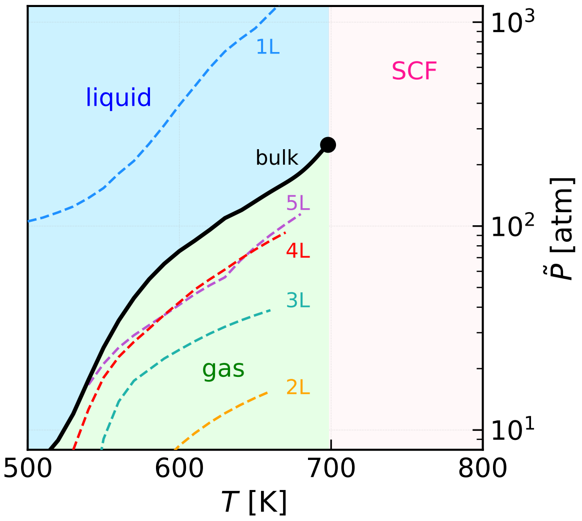

Fig. S9. Phase diagram of SCAN water upon confinement between graphene sheets. Liquid–vapor phase diagram in the $\tilde{P}$–T plane, for the different H indicated.

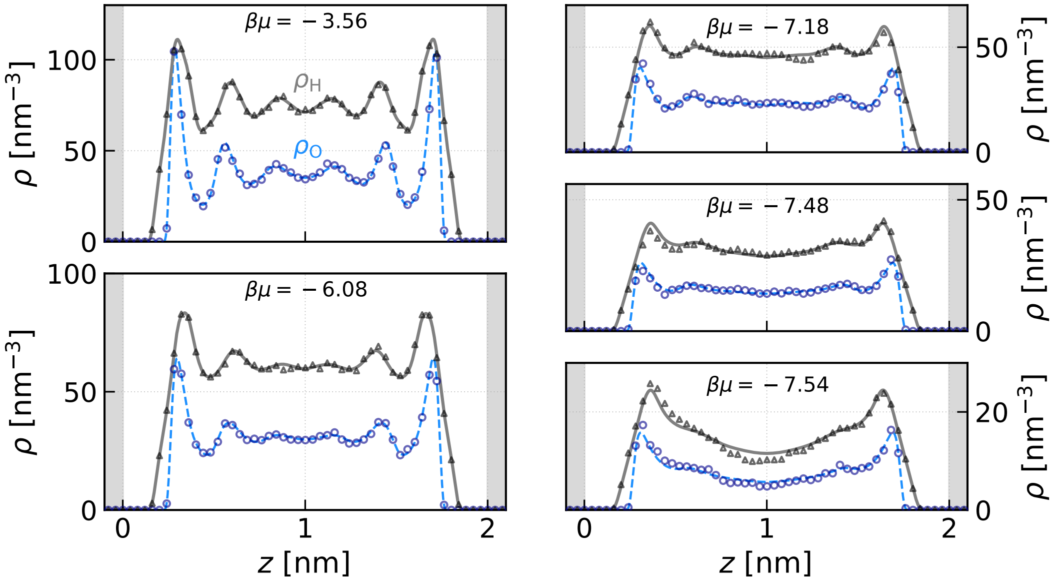

Fig. S10. Water's hydrogen atom density profiles obtained with hyper-DFT. For SCAN water confined between two graphene sheets at different chemical potentials at $T = 700$ K, we show the oxygen atom density profiles $\rho_{\mathrm{O}} = \rho$ obtained from neural cDFT (dashed blue line) and hydrogen atom density profiles ρ_{H} obtained from neural hyper-DFT (solid gray line), in good agreement with MD simulation (symbols).

References

1. L Zhang, J Han, H Wang, R Car, W E, Deep potential molecular dynamics: A scalable model with the accuracy of quantum mechanics. *Phys. Rev. Lett.* **120**, 143001 (2018).
2. J Behler, M Parrinello, Generalized neural-network representation of high-dimensional potential-energy surfaces. *Phys. Rev. Lett.* **98**, 146401 (2007).
3. I Batatia, DP Kovacs, G Simm, C Ortner, G Csanyi, MACE: Higher order equivariant message passing neural networks for fast and accurate force fields in *Adv. Neural Inf. Process. Syst.*, eds. S Koyejo, et al. (Curran Associates, Inc.), Vol. 35, pp. 11423–11436 (2022).
4. R Mathur, MC Muniz, S Yue, R Car, AZ Panagiotopoulos, First-principles-based machine learning models for phase behavior and transport properties of CO_2. *J. Phys. Chem. B* **127**, 4562–4569 (2023).
5. J Hansen, I McDonald, *Theory of Simple Liquids: with Applications to Soft Matter.* (Elsevier Science), (2013).
6. R Evans, U Marini Bettolo Marconi, Phase equilibria and solvation forces for fluids confined between parallel walls. *J. Chem. Phys* **86**, 7138–7148 (1987).
7. F Sammüller, M Schmidt, R Evans, Neural density functional theory of liquid-gas phase coexistence. *Phys. Rev. X* **15**, 011013 (2025).
8. F Sammüller, S Robitschko, S Hermann, M Schmidt, Hyperdensity functional theory of soft matter. *Phys. Rev. Lett.* **133**, 098201 (2024).
